# Establishment and Characterization of Immortalized Human Dermal Papilla Cells Expressing Human Papillomavirus 16 E6/E7

**DOI:** 10.4014/jmb.2310.10035

**Published:** 2023-11-17

**Authors:** Seonhwa Kim, Kyeong-Bae Jeon, Hyo-Min Park, Jinju Kim, Chae-Min Lim, Do-Young Yoon

**Affiliations:** Department of Bioscience and Biotechnology, Konkuk University, Seoul 05029, Republic of Korea

**Keywords:** Human dermal papilla cell, immortalization, human papillomavirus 16, E6/E7, Wnt/β-catenin, hair follicle formation

## Abstract

Primary human dermal papilla cells (HDPCs) are often preferred in studies on hair growth and regeneration. However, primary HDPCs are limited by their reduced proliferative capacity, decreased hair induction potential, and extended doubling times at higher passages. To overcome these limitations, pTARGET vectors containing human papillomavirus16 (HPV16) E6/E7 oncogenes were transfected into HDPCs and selected using G-148 to generate immortalized cells here. HPV16 E6/E7 oncogenes were efficiently transfected into primary HDPCs. Immortalized HDPC showed higher proliferative activity than primary HDPC, confirming an increased proliferation rate. Expression of p53 and pRb proteins was downregulated by E6 and E7, respectively. E6/E7 expressing HDPC cells revealed that cyclin-dependent kinase (CDK) inhibitor p21 expression was decreased, while cell cycle-related genes and proteins (CDK2 and cyclin E) and E2F family genes were upregulated. Immortalized HDPCs maintained their responsiveness to Wnt/β-catenin pathway and hair follicle formation capability, as indicated by their aggregative properties and stemness. E6/E7 immortalized HDPCs may facilitate *in vitro* hair growth and regeneration studies.

## Introduction

Hair dermal papilla cells (HDPCs) derived from the mesenchyme reside at the base of hair follicles. HPDCs play a crucial role in hair growth and development through interactions with the epithelial cells in hair follicles [1, 2]. The functions of HDPCs should be preserved to support continuous hair growth and development. However, cultured primary HDPCs lose hair inductive and proliferative capacity at higher passages [3]. Thus, establishing immortalized HDPCs that retain the properties of primary HDPCs is necessary for hair growth and regenerative research.

Transfection of cell lines with HPV16 genes, as in HPV infection of normal epithelial cells or transduction with lentiviral vectors harboring HPV E6/E7 genes, is an established method for cell line immortalization [4]. HPV16 E6/E7 oncogenes have been used to immortalize various cell lines, including human ameloblastoma, human oral epithelial, and bovine mammary epithelial cells [5-7]. HPV is a small, non-enveloped, double-stranded DNA virus that encodes early (E1, E2, E4, E5, E6, and E7) and late (L1 and L2) proteins [8]. Of these proteins, E6 and E7 oncoproteins from high-risk HPV promote cellular proliferation by deactivating p53 and retinoblastoma (Rb), respectively [9, 10]. E6 plays a central role in cell line immortalization by forming a ternary complex with the ubiquitin ligase E6 associated protein (E6AP) and p53 to trigger ubiquitination and subsequent degradation of p53 [11]. E7 is primarily located in the nucleus, where it binds and phosphorylates pRb, resulting in the release of E2F [12]. Immortalized HDPCs can be established by inactivating these major tumor suppressor proteins, p53 and pRb, through E6/E7 transfection [13].

Here, we transfected HPV16 E6/E7 oncogenes into early-passage primary HDPCs to generate immortalized HDPCs. We also performed an *in vitro* characterization of the immortalized HDPCs in terms of their proliferative capacity, Wnt/β-catenin signaling pathway activity, and hair follicle formation capability.

## Materials and Methods

### Reagents

5α-Dihydrotestosterone (DHT) solution was purchased from Sigma-Aldrich (#D-073, USA).

### Cell Culture

Primary human follicular dermal papilla cells (HDPCs) were obtained from PromoCell GmbH (Germany). The cells were cultured in hair follicle dermal papilla cell growth medium supplemented with 40 μl/ml of fetal calf serum, 4 μl/ml of bovine pituitary extract, 1 ng/ml of basic fibroblast growth factor (bFGF), and 5 μg/ml of insulin. The HDPCs were incubated at 37°C and 5% CO_2_. The cells were subjected to a maximum of 10 passages.

### Transfection of HPV16 E6E7

pTARGET/E6E7 was prepared as previously described [14]. In short, E6/E7 derived from HPV16 were amplified by PCR from total RNA isolated from CaSki cell lines using a following primer set: 5’-GCG GCC GCC ACC ATG TTT CAG GAC CAC AG-3’ (Forward) and 5’-AGG CGG CCG CGA TTA TGG TTT CTG AGA ACA-3’ (Reverse). The PCR products were inserted into pCR2.1-TOPO cloning vector (Invitrogen, USA) and digested with EcoRI. The excised E6/E7 vector was ligated into the pTARGET vector (Promega) to construct pTARGET/E6E7. We transfected the pTARGET/E6E7 construct (5 μg/well) into primary HDPCs using the OmicsFect *in vitro* transfection reagent (OmicsBio, Taiwan). Twenty-four hours after transfection, 500 μg/ml of G-418 was added to the wells in the transfection group and incubated for an additional 24 h. Only G-418 selected cells were used.

### Cell Viability Assay Using MTS Assay

Cell proliferation rate was estimated using the 3-(4,5-dimethylthiazol-2-yl)-5-(3-carboxymethoxy phenyl) -2-(4-sulfophenyl)-2H-tetrazolium (MTS) assay with a CellTiter 96 Aqueous One Solution Assay (Promega, USA). Primary and immortalized HDPCs (1.0 × 10^4^ cells/well) were seeded overnight in 100 μl of the medium in 96-well plates and then incubated for 0, 24, 48, and 72 h. The reagent (20 μl) was added to each well and incubated for 30 min. The absorbance was measured at 492 nm using a microplate reader (Apollo LB 9110; Berthold Technologies GmbH, Bad Wildbad, Germany).

### RNA Isolation and Reverse-Transcription PCR (RT-PCR)

Primary and immortalized HDPCs were seeded in 6-well plates. When the cells reached 100% confluence, they were collected and lysed using an easy-BLUE Total RNA Extraction Kit (iNtRon Biotechnology, South Korea). For reverse-transcription polymerase chain reaction (RT-PCR), RNA was reverse-transcribed to cDNA using oligo (dT) primers and M-MuLV reverse transcriptase (New England Biolabs, USA). RNA was reverse transcribed into cDNA using Prostar (Stratagene, USA). The synthesized cDNA was amplified using a PCR Thermal Cycler Dice (Takara, Japan). The primers used in this study are listed in Table 1.

### Immunoblotting Assay

Primary and immortalized HDPCs were seeded in 6-well plates and incubated until they reached 100%confluence. Harvested cells were washed with phosphate-buffered saline (PBS) and lysed in buffer containing 150 mM NaCl, 0.1% sodium dodecyl sulfate, 0.25% sodium deoxycholate, 1 mM ethylene diamine tetra-acetic acid, 1% NP40, 0.4 mM phenylmethylsulfonyl fluoride, 1 mM orthovanadate, 1 mM ethylene glycol tetra-acetic acid, 50 mM Tris (pH 7.4), and aprotinin (10 μg/ml) at 4°C for 30 min. Equal amounts of the quantified proteins were separated by sodium dodecyl sulfate-polyacrylamide gel electrophoresis (SDS-PAGE) and transferred onto polyvinylidene difluoride membranes. Specific primary antibodies against p53 (#NB200-171) (Novus Biologicals, Centennial, USA), cyclin E (#sc-481) (Santa Cruz Biotechnology, USA), p21(#2947T), CDK2 (#2546T), pRb (#9309s), and p-pRb (#8147s) (Cell Signaling Technology, USA) were used. Densitometric graphs of three independent experiments were generated using ImageJ software (version 1.5) [15]. Band intensities were normalized to those of β-actin or GAPDH.

### Immunofluorescence Staining

Primary and immortalized HDPCs were seeded in 8-well cell culture slides and incubated for another 24 h. Paraformaldehyde (4%) and methanol (100%) were used for cell fixation and permeabilization, respectively. BSA in PBS (1%) was used to block nonspecific binding of antibodies. Specific antibodies against p53 (1:2,000 dilution), pRb (1:200 dilution), and β-catenin (1:2000 dilution) (Sigma) were incubated overnight at 4°C. The cells were then incubated with goat anti-rabbit or anti-mouse IgG secondary antibodies labeled with Cy3 or FITC (Merck Millipore, Darmstadt, Germany, 1:400 dilution) for 1 h. The cells were stained with 4,6-diamidino-2-phenylindole (DAPI) (1:1,000 dilution) (Sigma). A confocal fluorescence microscope (EVOSTM M7000; Thermo Fisher Scientific Inc., USA) with a 20× objective was used to obtain fluorescence images.

### RGB Profiling of Immunofluorescence Images

Images were prepared to include a legend with a scaling bar to represent actual cell sizes. The scale was adjusted using the ImageJ software. Briefly, a representative cell was selected and a line was drawn at the location that passed through the nucleus. The fluorescence intensity along the drawn line was measured using the red-green-blue (RGB) profiler of ImageJ software. This generates a single plot that shows the profile of an RGB image in three different colors (red, green, and blue).

### Evaluation of 3D Spheroid Construction Ability

To assess whether HDPCs induce the 3D spheroid, primary and immortalized HDPCs (1.5 × 10^5^ cells/ml) were seeded in a 96-well round-bottom cell floater plate. The cells were incubated for 48 h to generate spheroids. Images of the 3D spheroids were obtained using a phase-contrast microscope. The average value of each spheroid was estimated by measuring the diameter five times using the ImageJ software.

### Statistical Analysis

Data were presented as the mean standard deviation (SD) of three independent experiments. GraphPad Prism 9 software (GraphPad Software Inc., USA) was used for the statistical analysis. Unpaired *t*-tests were used. Statistical significance was defined as follows: *p < 0.05, **p < 0.01, ***p < 0.001, and ****p < 0.0001.

## Results

### Detection of HPV16 E6/E7 in HDPCs

**Fig. 1:**
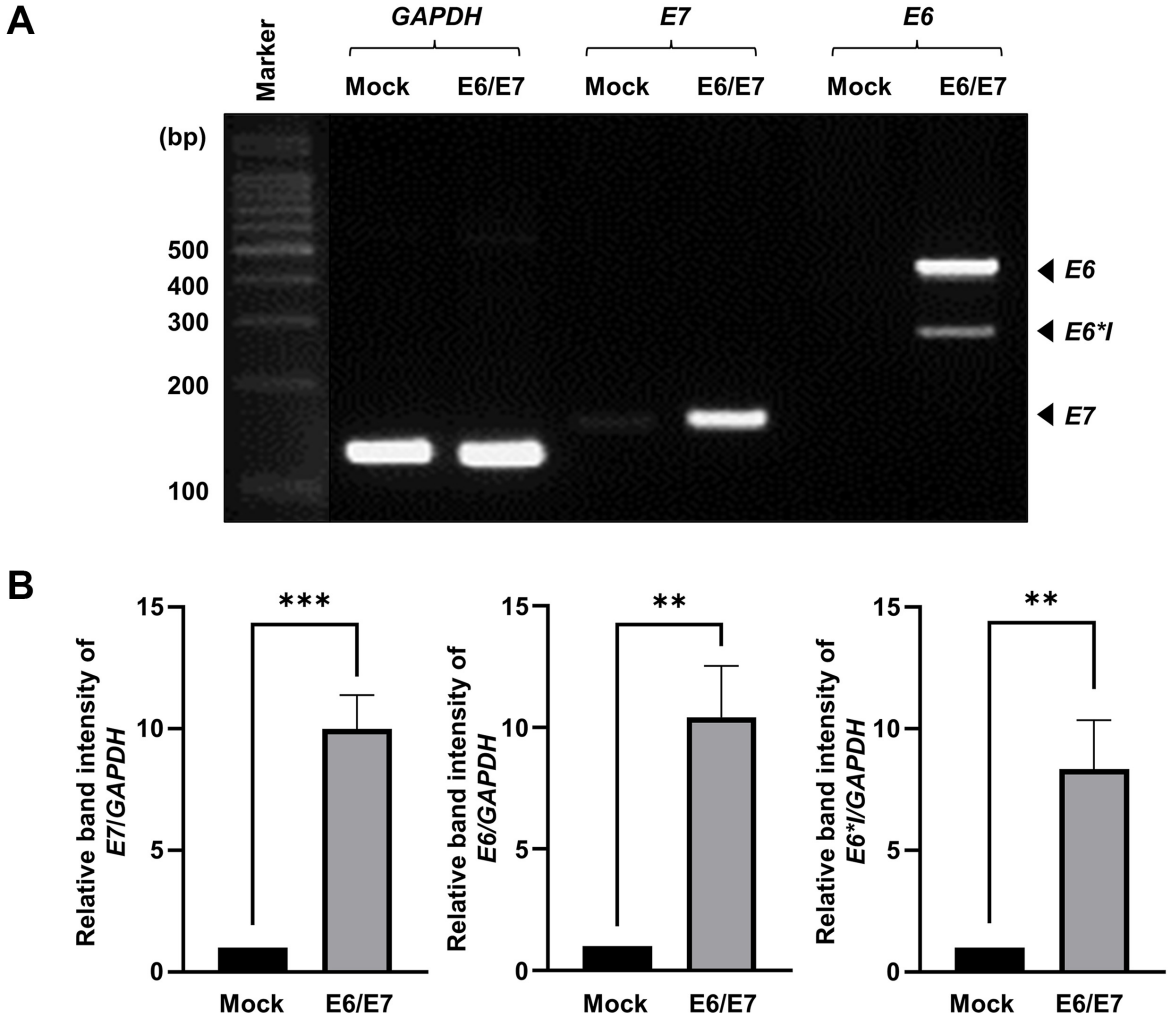
Quantification of E6 and E7 expression in HDPCs. (**A**) Expression levels of E6, E7, and GAPDH were determined. (**B**) The relative band intensities of E6, E6*I and E7 to GAPDH were assessed by ImageJ software. Data are exhibited as the mean SD (*n* = 3). **p < 0.01, ***p < 0.001 by *t*-test.

The primary HDPC cells were stably transfected with either E6/E7 from the integrated HPV16 genome or a mock vector, and the effect of E6/E7 on cell cycle progression was investigated. RT-PCR was used to quantify the expression of E6 and E7 in the respective transfectants (Fig. 1A and 1B). HPV16 E6 and E7 mRNA levels were significantly higher in immortalized HDPCs compared to primary HDPCs. Most E6 transcripts were full-length E6, and the spliced form E6*I was detected at low levels.

### The Effect of E6/E7 on Proliferation and Morphology in HDPCs

**Fig. 2:**
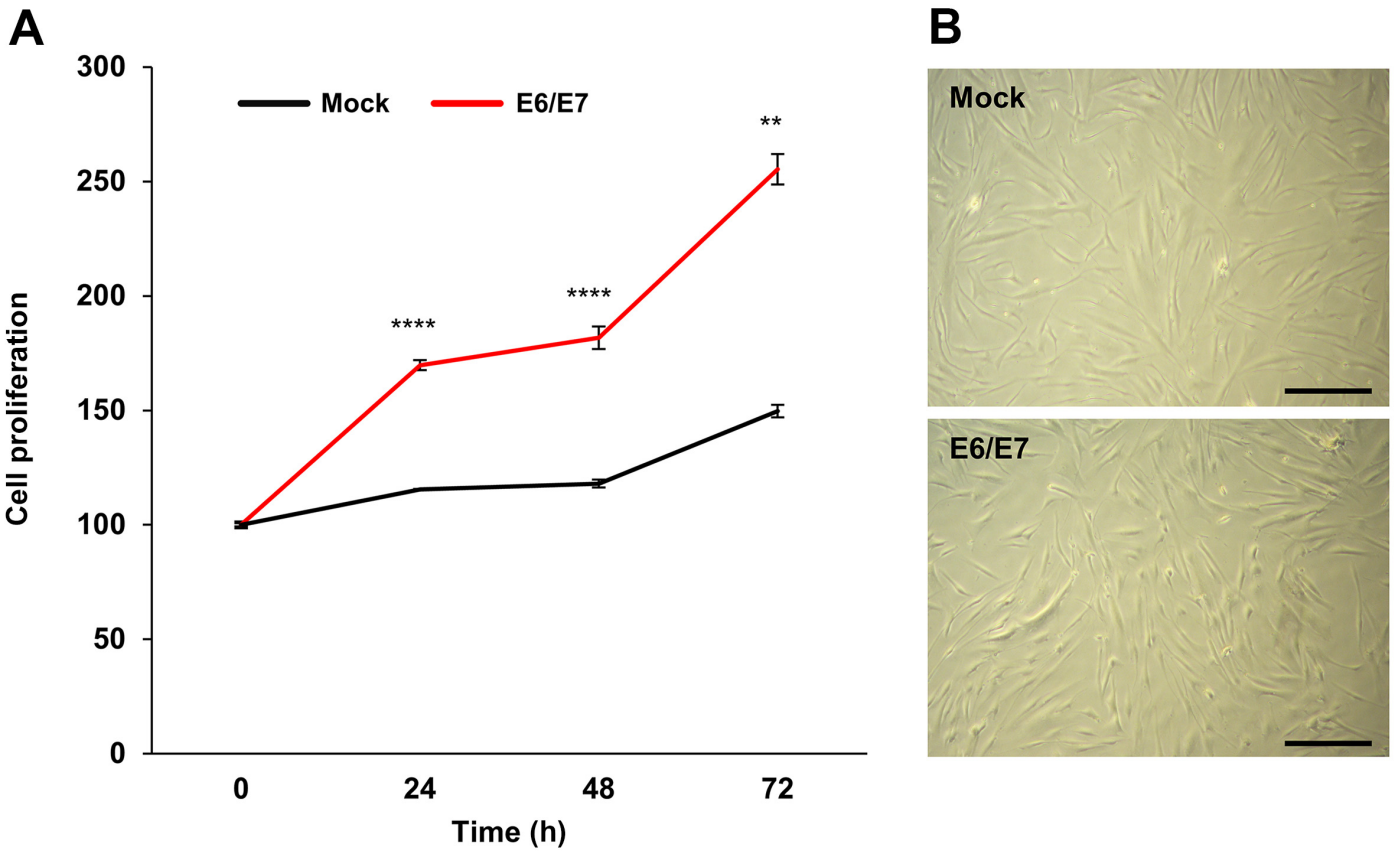
Comparison of cell growth and morphology between primary and immortalized HDPCs. (**A**) Cell growth curves of primary and E6/E7 immortalized HDPCs. Data are exhibited as the mean SD (*n* = 3). **p < 0.01, ****p < 0.0001 by *t*-test. (**B**) Comparison of cell morphological features between primary and immortalized HDPC. Cell morphologies were confirmed by an inverted phase contrast microscope (magnification at × 4). Scale bar = 500 μm.

The proliferation rates of primary and immortalized HDPCs were determined using the MTS assay. The rate was measured every 24 h for 72 h. The proliferation rates of primary and immortalized HDPCs were 149.7% and 255.3%, respectively. Hence, HPV16 E6/E7 immortalized HDPCs proliferated at a 1.71-fold higher rate than primary HDPCs under the same conditions (Fig. 2A). Both primary and immortalized HDPCs showed uniform cell sizes and did not show any differences from each other (Fig. 2B). These results indicate that E6/E7 oncoproteins increase the proliferation rate without affecting cell characteristics.

### The Effect of E6 on Expression of p53 and Cell Cycle Related Factors

**Fig. 3:**
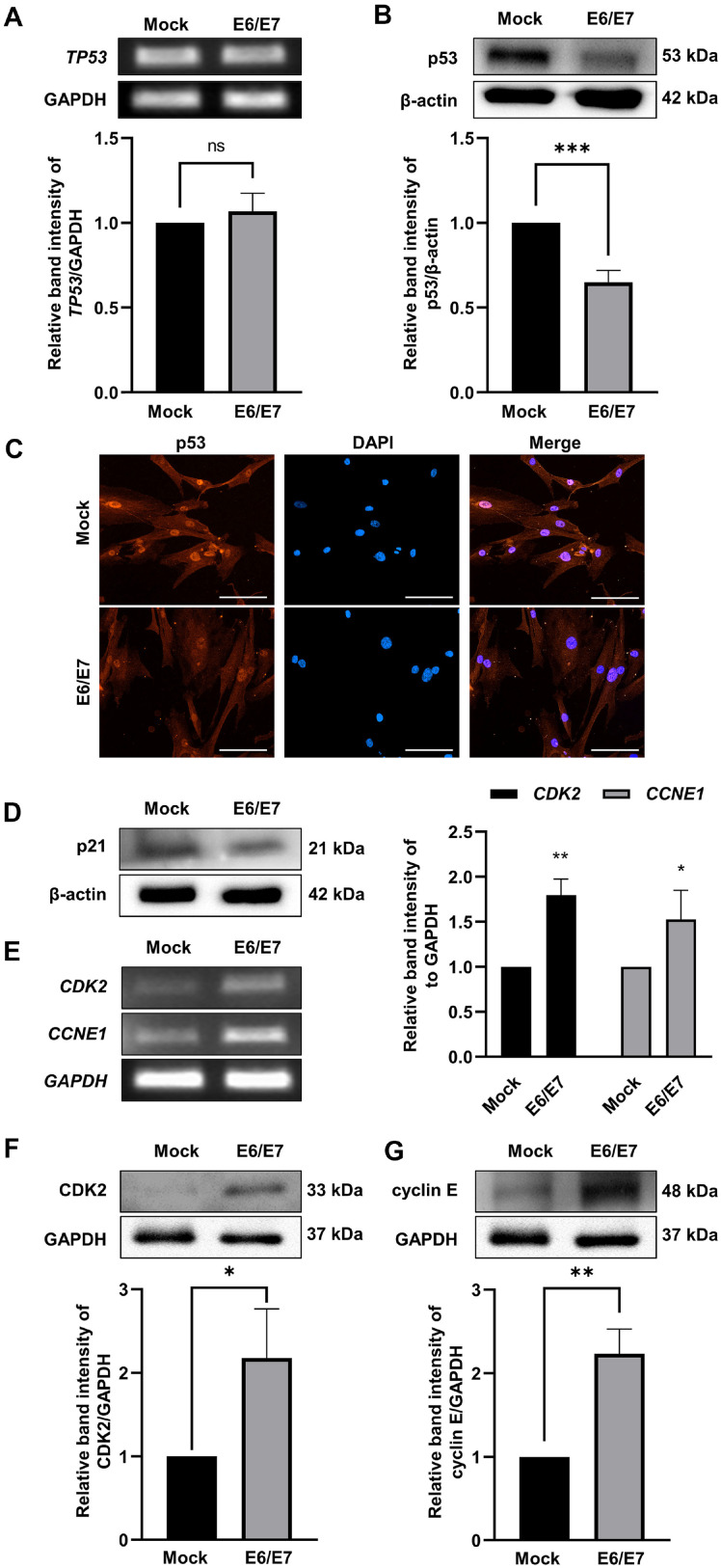
Expression levels of p53 and cell cycle related factors in HDPCs. (**A, B**) The TP53 gene and p53 protein expression level was determined. The relative band intensities were evaluated by ImageJ software. Data are presented as the mean SD (*n* = 3). ***p < 0.001 by *t*-test. (**C**) p53 was labeled with Cy3 (red) in cells, and nuclei were labeled with DAPI (blue). Scale bar = 125 μm. (**D**) The protein level of CDK inhibitor p21 was determined by immunoblotting assay. (**E-G**) The gene (CDK2 and CCNE1) and protein (CDK2 and cyclin E) expression levels were determined. The relative band intensities were evaluated by ImageJ software. Data are presented as the mean SD (*n* = 3). *p < 0.05, **p < 0.01 by *t*-test.

First, p53 levels were quantified in primary and immortalized HDPCs. Gene expression levels of TP53 were not altered (Fig. 3A), whereas expression of p53 protein was downregulated (Fig. 3B). Downregulation of p53 protein expression in immortalized HDPCs, especially in the nucleus, was also confirmed using immunofluorescence staining (Fig. 3C). We then assessed the effect of E6/E7 on the cyclin-dependent kinase (CDK) inhibitor p21. p21 protein expression was downregulated in immortalized HDPCs (Fig. 3D). CDK2 and CCNE1 mRNA levels were upregulated in immortalized HDPC (Fig. 3E). Protein expression levels of CDK2 and cyclin E were also increased in E6/E7 immortalized HDPCs (Fig. 3A and 3B). These results indicate that p53 is degraded through the proteasome via E6, leading to the decrease in p21 expression, and increase in CDK and cyclin E.

### The Effect of E7 on Expression Levels of pRb and Transcription Factor E2F

**Fig. 4:**
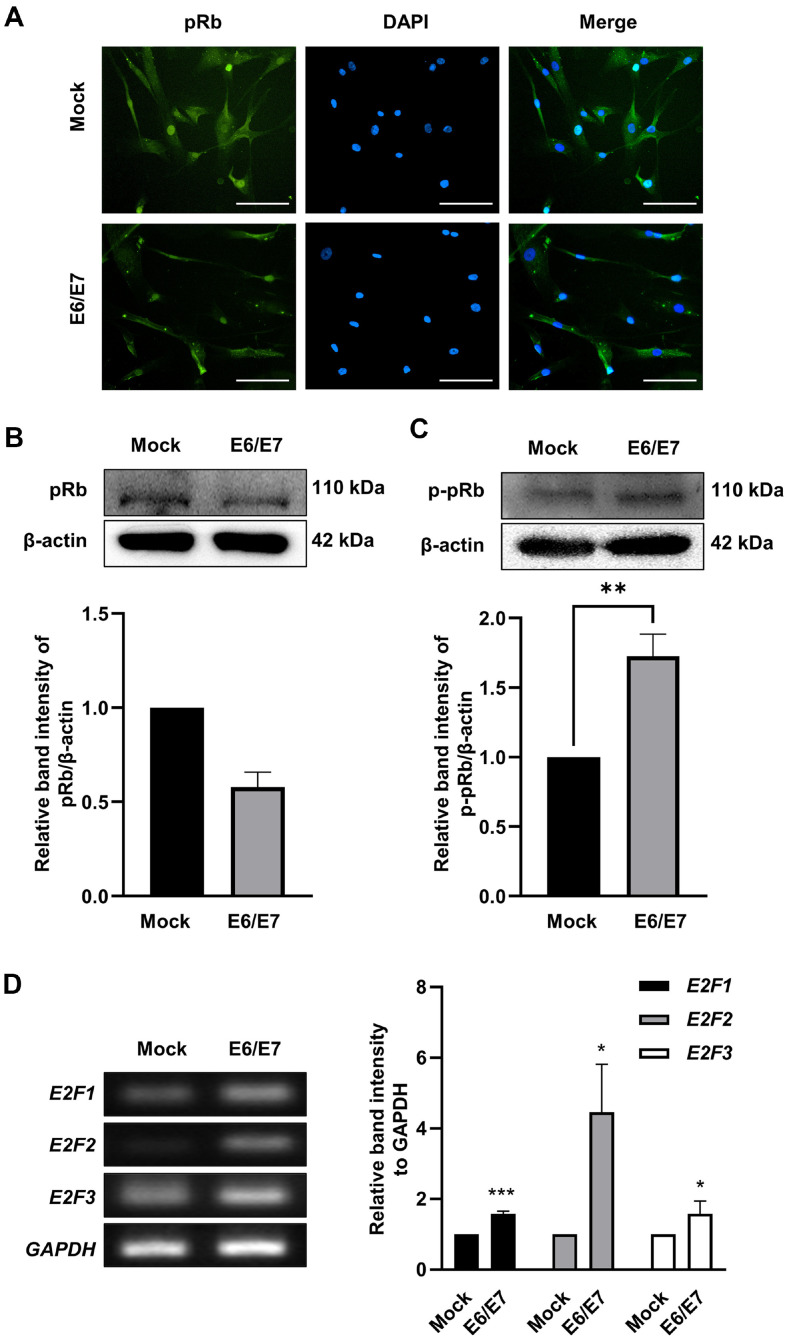
Expression levels of pRb protein and E2F genes in HDPCs. (**A**) pRb was labeled with FITC (green) in cells, and nuclei were labeled with DAPI (blue). Scale bar = 125 μm. (**B, C**) The pRb and p-pRb protein level was determined by immunoblotting assay. The relative band intensities were evaluated by ImageJ software. Data are presented as the mean SD. **p < 0.01 by *t*-test. (**D**) The gene expression levels of E2F1-3 were determined. The relative band intensities were evaluated by ImageJ software. Data are reported as the mean SD (*n* = 3). *p < 0.05, **p < 0.01, ***p < 0.001 by *t*-test.

Next, the direct effects of E7 on pRb and E2F expression in HDPCs were evaluated. Immunofluorescence staining revealed that pRb expression was downregulated in immortalized HDPCs, especially in the nucleus (Fig. 4A). The reduction in pRb expression and increase in p-pRb levels in immortalized HDPCs were also confirmed by immunoblotting assays (Fig. 4B and 4C). In addition, mRNA levels of E2F factors, including E2F1, E2F2, and E2F3, were increased in immortalized HDPCs (Fig. 4D). These results indicate that E7 hyperphosphorylates pRb, leading to the release of E2F which promotes transcription of genes involved in G1-S transition.

### Responsiveness to Wnt/β-Catenin Signaling and Hair Follicle Induction in E6/E7 Immortalized HDPCs

**Fig. 5:**
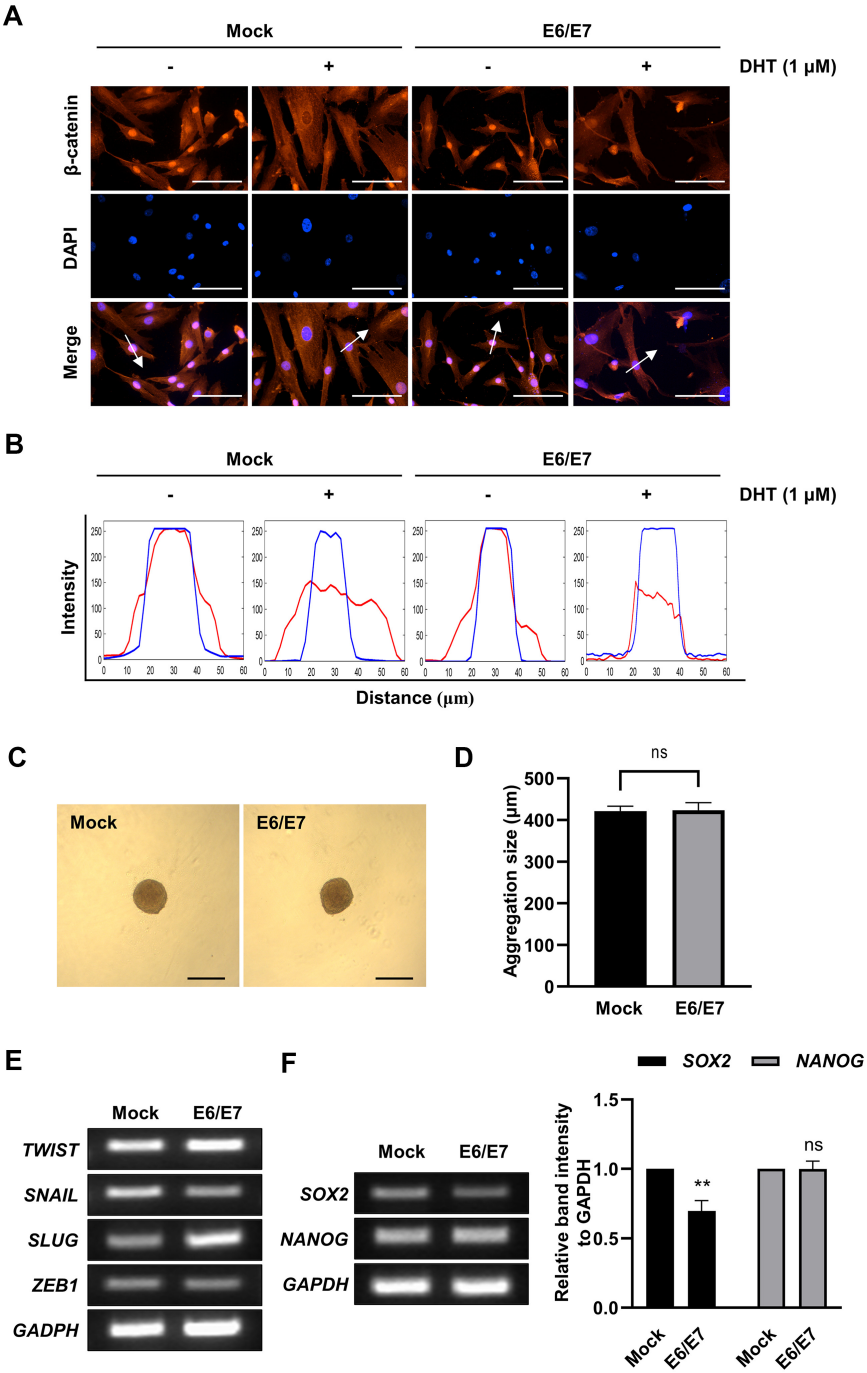
Responses to Wnt/β-catenin signaling and hair follicle formation capability in HDPCs. (**A**) β-catenin was labeled with Cy3 (red) in cells, and nuclei were labeled with DAPI (blue). Scale bar = 125 μm. (**B**) Fluorescence intensity was profiled (white arrow sign on third line of (**A**)). (**C, D**) Representative image of 3D spheroid was confirmed (magnification at × 4). Scale bar = 500 μm. The size in diameter of spheroids were quantified by ImageJ software. (**E, F**) The gene expressions of EMT markers (TWIST, SNAIL, SLUG, and ZEB1) and self-renewal markers (SOX2 and NANOG) were estimated. The relative band intensities were evaluated by ImageJ software. Data are reported as the mean SD (*n* = 3). **p < 0.01 by *t*-test.

Primary and immortalized HDPCs were treated with DHT (1 μM) to assess their Wnt/β-catenin pathway responsiveness. DHT treatment for 2 h inhibited the nuclear translocation of β-catenin in both HDPC types (Fig. 5A). By RGB line profiling, it was also confirmed that β-catenin intensity in the nucleus was low (Fig. 5B). These findings indicate that immortalized HDPCs retained their responsiveness to the Wnt/β-catenin signaling pathway.

Since the induction of hair follicles is related to the aggregation of HDPCs, we also investigated their 3D spheroid formation capability of the transfected cells. Both primary and immortalized HDPCs formed round and compact 3D spheroids with a size of approximately 420 μm (Fig. 5C and 5D). To confirm whether E6/E7 affects the stemness of HDPCs, epithelial-mesenchymal transition (EMT) and self-renewal markers were quantified. EMT markers were well expressed even in the immortalized HDPCs (Fig. 5E). Immortalized HDPCs showed a slight decrease in SOX2 expression, whereas there were no noticeable differences in NANOG expression (Fig. 5F). These results indicated that both the aggregation ability and stem cell-like characteristics were preserved in immortalized HDPCs.

## Discussion

In this study, we established and characterized immortalized HDPCs via E6/E7 transfection. E6 and E7 oncoproteins from high-risk HPV16 inhibit p53 and pRb, respectively, and thereby enable cellular immortalization [16]. Full-length E6 open reading frames (ORF) (450 bp) and truncated E6 ORFs including E6*I (300 bp) and E6*II (150 bp) are generated by splicing of HPV16 E6 [17]. Full-length E6, truncated E6*I, and E7 were detected only in immortalized HDPC (Fig. 1). Moreover, immortalized cells showed a 1.71-fold higher proliferation rate than primary cells, with no morphological changes (Fig. 2).

HPV E6*I binds p53 to induce proteasomal degradation of p53, which may result in cell immortalization [18]. p53 regulates cellular responses including DNA repair, cell cycle arrest, apoptosis induction, and metabolism [19-22]. TP53 expression did not differ between primary and immortalized HDPCs (Fig. 3A). However, p53 protein levels were lower in immortalized HDPCs compared to primary HDPCs (Fig. 3B-3C). These results indicate that p53 underwent increased proteasomal degradation through binding to the E6 protein. The CDK inhibitor p21 is the transcriptional target of p53. When activated, p53 binds the p21 promoter to increase transcription [23, 24]. As expected, p21 protein expression was decreased due to the downregulated p53 (Fig. 3D). p21 regulates cell cycle progression by inhibiting CDK proteins, which form complexes with cyclin to control various cell cycle stages [25]. The G1-S phase transition is associated with CDK2, which is activated by cyclin E1 and E2 [26, 27]. CDK2 and CCNE1 mRNA levels and CDK2 and cyclin E protein levels were significantly upregulated in immortalized HDPCs (Fig. 3E-3G). These upregulations promote G1-S transition and induce cell proliferation.

E7 in high-risk HPVs has a higher affinity for pRb than E7 in low-risk HPVs, leading to a reduction in pRb level [28]. E7 interacts with retinoblastoma (Rb) family proteins, such as pRb, p107, and p130 [29]. pRb expression was decreased in immortalized HDPCs (Fig. 4A-4B). However, p-pRb expression was increased compared to primary HDPCs (Fig. 4C), which was due to the increase in CDK and cyclin E as shown in Fig. 3E-3F. Upon formation in G1, cyclin E-CDK2 complexes translocate to the nucleus and phosphorylate pRb, effectively inactivating it [30]. Free E2F regulates the expression of genes, including cyclin, CDK, and E2F themselves, which regulate the G1-S transition [26, 31]. Only E2F1-3 among the E2F family bind pRb, and its overexpression efficiently induces G1-S transition [28, 32]. We measured E2F1, E2F2, and E2F3 mRNA levels in the two types of HDPCs and found significant upregulation in immortalized HDPCs (Fig. 4D). This upregulation was expected to increase the expression of genes critical for G1-S transition.

As a potent androgen, DHT plays a key role in regulating the hair cycle. Hair cycling relies on crosstalk between the androgen and Wnt/β-catenin pathways [33]. Wnt/β-catenin pathway is active in the dermal papilla of growing hair follicles during the anagen phase, and maintaining the activity of this signaling pathway is essential for preserving cultured HDPCs in a state that promote hair growth [34]. β-catenin activity was inhibited by DHT treatment (Fig. 5A and 5B), confirming that the response to Wnt/β-catenin signaling pathway sustained functional even in immortalized HDPCs.

HDPCs are multipotent stem cells and their aggregative properties and stemness are associated with hair follicle formation [35, 36]. 3D spheroids efficiently induce HDPC that can form hair follicles in the skin [37]. Previous studies demonstrated that 3D spheroids of HDPCs form *de novo* hair follicles on mice skin [38]. Models with co-cultures incorporating both dermal papilla cells and keratinocytes have also been developed, providing evidence that their co-cultivation effectively preserves the in vivo properties of dermal papilla cells [39, 40]. The human folliculoid microsphere, consisting of HDPC and outer root sheath keratinocytes, has also been reported. This model demonstrated close interaction between the two types of cells and expressed versican, which is essential for hair follicle formation [41]. In addition, maintenance of the stem cell-like characteristics is crucial to maintain HDPC functionality. EMT and self-renewal factors are markers of stemness in HDPCs [35]. Transcription factors such as Twist, Snail, and Slug are found in cells undergoing EMT and play a significant role in preserving stem cell function [42, 43]. Self-renewal, a key characteristic of stem cells, is facilitated by SOX2 and NANOG [44]. SOX2 is a well-known HDPCs marker [45]. Thus, the aggregative behavior, EMT markers (TWIST, SNAIL, SLUG, and ZEB1), and self-renewal markers (SOX2 and NANOG) were evaluated in both cell types. E6/E7 transfection did not affect the aggregation or stemness of HDPCs (Fig. 5C-5F). This suggests that immortalized HDPCs retained their ability to form hair follicles while maintaining their characteristic features.

## Conclusion

Here, we established and characterized immortalized HPDC cells by transfecting them with the HPV 16 E6/E7 oncogenes. Upon transfection, proliferation rate of cells was promoted through regulating of p53 and pRb activity. The cells also exhibited preserved responsiveness to the Wnt/β-catenin pathway, and retained their ability to form hair follicles, as demonstrated by their aggregating properties and stem cell characteristics. Immortalized HDPCs may be useful for *in vitro* research on mechanisms of hair growth and regeneration.

## Figures and Tables

**Table 1:** Primer sequences and product sizes used in RT-PCR.

Gene	Primer	Sequence (5’-3’)	Product Size (bp)
E6	Forward	ATG TTT CAG GAC CCA CAG GA	456
	Reverse	TTA CAG CTG GGT TTC TCT ACG	
E7	Forward	ATG CAT GGA GAT ACA CCT AC	200
	Reverse	AAC CGA AGC GTA GAG TCA CAC	
E2F1	Forward	AAG GCC CGA TCG ATG TTT TC	51
	Reverse	TGA TCC CAC CTA CGG TCT CCT	
E2F2	Forward	GAG GAC AAG GCC AAC AAG AGG	62
	Reverse	TTG CCA ACA GCA CGG ATA TC	
E2F3	Forward	GAA TAT CCC TAA ACC CGC TTC	51
	Reverse	TGT CCT GAG TTG GTT GAA GCC	
CDK2	Forward	GCT ATC TGT TCC AGC TGC TC	134
	Reverse	CTG GCT AGT CCA AAG TCT GC	
CCNE1	Forward	GTT ATA AGG GAG ACG GGG AG	205
	Reverse	TGC TCT GCT TCT TAC CGC TC	
TWIST	Forward	GCC AGG TAC GAC TTC CTC T	122
	Reverse	TCC ATC CTC CAG ACC GAG AAG G	
SNAIL	Forward	TGC CCT CAA GATGCA CAT CCG A	133
	Reverse	GGG ACA GGA GAA GGG CTT CTC	
SLUG	Forward	ATC TGC GGC AAG GCG TTT TCC A	127
	Reverse	GAG CCC TCA GAT TTG ACC TGT C	
ZEB1	Forward	GCC AAT AAG CAA ACG ATT CTG	101
	Reverse	TTT GGC TGG ATC ACT TTC AAG	
SOX2	Forward	CAT GAA GGA GCA CCC GGA TT	189
	Reverse	TTC ATG TGC GCG TAA CTG TC	
NANOG	Forward	ACC AGT CCC AAA GGC AAA CA	166
	Reverse	TCT GCT GGA GGC TGA GGT AT	
GAPDH	Forward	AGA ACA TCA TCC CTG CCT CT	131
	Reverse	CTG CTT CAC CAC CTT CTT GA	
